# Assessing the quality of life in the families of patients with epidermolysis bullosa: The mothers as main caregivers

**DOI:** 10.1016/j.ijwd.2021.08.007

**Published:** 2021-08-26

**Authors:** Fatemeh Chogani, Mohammad Mahdi Parvizi, Dedee F. Murrell, Farhad Handjani

**Affiliations:** a Student Research Committee, Shiraz University of Medical Sciences, Shiraz, Iran; b Molecular Dermatology Research Center, Shiraz University of Medical Sciences, Shiraz, Iran; c The George Institute for Global Health, University of New South Wales, Sydney, Australia; d Department of Dermatology, School of Medicine, Shiraz University of Medical Sciences, Shiraz, Iran

**Keywords:** Quality of life, epidermolysis bullosa, skin disease

## Abstract

**Background:**

Epidermolysis bullosa (EB) is an uncommon group of inherited disorders characterized by skin blistering after friction or mechanical trauma. EB affects patients and their families physically, socially, and emotionally.

**Objective:**

This study aimed to assess the family quality of life of these patients using the Family Dermatology Life Quality Index (FDLQI) questionnaire.

**Methods:**

In this cross-sectional study, we enrolled caregivers of patients with EB registered at the Molecular Dermatology Research Center, affiliated with Shiraz University of Medical Sciences, up to 2020. Participants filled out a demographic data collection form and the FDLQI questionnaire. The data were analyzed using SPSS software, version 22.

**Results:**

Overall, 80 participants, consisting of 65 mothers (81.2%) and 15 fathers (18.7%) as primary caregivers, were enrolled in this study. The average FDLQI score was 19.88 ± 4.71. The FDLQI scores of caregivers of patients with EB simplex was significantly lower than scores observed in those with other types of EB (*p* < .001). There was a significant positive association between the number of patients with EB in the family and FDLQI score (*p* = .049). FDLQI scores were lower in caregiving mothers who had a higher education (*p* < .001) and those who were employed (*p* < .001).

**Conclusion:**

Family quality of life is affected in patients with EB. Families with lower socioeconomic status and unemployed caregivers require special attention. More studies are needed to determine the parameters involved in the quality of life of patients with EB and their families.

**What is known about this subject in regard to women and their families?**

• Overall, the quality-of-life scores of patients with dystrophic epidermolysis bullosa are higher than those with other types of this condition.
• The families of patients with epidermolysis bullosa have to endure the high costs involved with the medical care of their children.
• Very few studies from Iran have addressed the problems faced by patients with epidermolysis bullosa and their families in dealing with this genetic disorder.

**What is new from this article as messages for women and their families?**

• Mothers were the main caregivers for patients with epidermolysis bullosa in Iran.
• Employed mothers and mothers with a higher education had a better quality of life.
• Mothers with comorbid diseases had a lower quality of life.

Alt-text: Unlabelled box

## Introduction

Epidermolysis bullosa (EB) is a rare condition characterized by fragility of the skin and mucous membranes (Fine and Mellerio, 2009; Intong and Murrell, 2012). Minimal mechanical trauma results in blister formation (Siañez-González et al., 2009) due to mutations that cause changes in tissue proteins (Oliveira et al., 2010). Based on a report by the National Epidermolysis Bullosa Registry during a period of 16 years across the United States, inherited EB accounts for 19.57 and 11.07 per 1 million live births and per 1 million population, respectively (Fine, 2016). Although there is no accurate estimate of the prevalence of EB in Iran, available data suggest that the proportion of affected cases, particularly recessive forms of EB, appears to be higher due to a higher number of consanguineous marriages (Parvizi et al., 2017).

Based on clinical presentation and histopathology assessment, EB is classified into four main subtypes: EB simplex with intraepidermal cleavage, junctional EB with intralamina lucida cleavage, recessive or dominant dystrophic EB with sublamina densa cleavage, and Kindler syndrome with intralamina lucida and sublamina densa cleavage.

Patients with EB may present with different acute or chronic and distinctly severe clinical manifestations. The clinical picture of EB covers a wide variety of manifestations, including pain and pruritus, ulcers, blistering, skin infections, microstomia, ankyloglossia, esophageal stricture, dysphagia and malnutrition, gastrointestinal erosions, pseudo-syndactyly, skin cancers, and scarring that leads to skin contracture in the axilla and knees, some of which subsequently alters walking and daily activities (Fine et al., 2014; Haynes, 2006; Zidorio et al., 2015). All of these mentioned manifestations can affect the patient's quality of life (QoL; Sebaratnam et al., 2012; Togo et al., 2020).

The QoL assessment of patients with EB is an important consideration for the management and control of this condition, given the complexity and chronicity of the disease. In this context, a specific instrument to assess the quality of life in patients with EB (QoL EB questionnaire) has been developed and is currently used for patient care and research purposes (Frew and Murrell, 2010; Frew et al., 2009).

Furthermore, EB has been shown to have a considerable clinical and socioeconomic impact on patients and their families (Jain and Murrell, 2018). In this regard, a general approach to evaluating the QoL of patients’ families or caregivers is required to evaluate the burden of the disease (Basra et al., 2007). Generic and specific QoL tools have been introduced for patients with dermatologic conditions (Horn and Tidman, 2002; Tabolli et al., 2009) and their families. (Basra et al., 2007; Ferrario et al., 2004). The Family Dermatologic Life Quality Index (FDLQI) is a basic dermatology-specific instrument that can be self-administered and used as an alternative outcome indicator in clinical practice and research (Basra et al., 2007). It may be used in conjunction with other health-status indicators to discuss concerns, such as health care funding and resource allocation within and between specialties. It assesses the effect of skin disorders on family members over the previous month. The FDLQI questionnaire has been translated into and validated in many languages, including the Persian language (Safizadeh et al., 2014). In addition, efforts have been undertaken to specifically assess the burden of disease on families of children with EB, and a specific questionnaire (Epidermolysis Bullosa Burden of Disease) has been developed (Dufresne et al., 2015).

The lives of children with EB are inextricably linked with those of their parents because finding and training suitable caregivers on the use of nonstick dressings is difficult; usually caregivers have to be nurses, which is expensive. Parents share this responsibility, but previous studies have shown that often one parent has to sacrifice their career aspirations to have time to provide the care needed. Parents rarely have sufficient income to pay for skilled care (Bruckner et al., 2020; Gorell et al., 2020; Manomy et al., 2021; Wu et al., 2020). Hence, we examined whether impact on family quality of life (FQoL) could be measured. The survey was conducted in Iran to find out who the main caregiver was and measure the FQoL to better understand problems that may exist and as a parameter to better manage the disease.

## Methods

### Study population

The study population included the caregivers of 80 patients with EB who had registered at the Molecular Dermatology Research Center, affiliated with Shiraz University of Medical Sciences in the Fars province in southwestern Iran, until October 2020.

### Data collection

In this cross-sectional study, the participants were entered into the study with the census method. The approach to assess FQoL in this study consisted of two parts. The first part was a questionnaire to gather demographic information about patients and their caregivers. For the second part, the FDLQI questionnaire, which has already been translated and validated in Persian, was used. The FDLQI questionnaire was developed at Cardiff University, and the authors received permission to use it (License ID: CUQoL2902; Safizadeh et al., 2014). The questionnaire consists of 10 questions based on the Likert scale. The answers are scored from 0 to 3, with a total score range between 0 and 30 points. A higher score indicates a lower FQoL.

Of note, our study was conducted during the COVID-19 pandemic; therefore, due to the risk of virus transmission, in cases where face-to-face interviewers could not be conducted, the information was gathered by calling the parents of the patients with EB.

### Ethical statements

Verbal informed consent was obtained from all participants. Individuals who did not agree to participate were excluded from the study. The completed questionnaires were also coded to keep the participants' names confidential. The study was approved by the ethics committee of Shiraz University of Medical Sciences (Approval ID: IR.SUMS.MED.REC.1399.375).

### Statistical analysis

The data were analyzed with SPSS, version 22. The data were first evaluated for normality with the Kolmogorov–Smirnov normality test. After confirmation of normal distribution, data analysis was conducted using independent sample Student *t* test, with an analysis of variance test for continuous and χ^2^ for categorical variables. Descriptive statistics were reported as frequency, percentage, mean data distribution, and standard deviation. Moreover, a multiple linear regression with a backward model was used to find the cofounding factors affecting the FDLQI scores.

## Results

Based on the number of questionnaires received, a total of 80 participants were enrolled in our study (one caregiver for each patient registered at our center). The patients' average age was 13.34 ± 10.37 years with a sex distribution of 47 boys (58.7%) and 33 girls (41.3%). The average FDLQI score was 19.88 ± 4.71 (out of 30). Table 1 depicts the FDLQI scores reported by the parents of patients with EB.

**Table 1:** Frequency of answers to the 10 items of the Family Dermatology Life Quality Index questionnaire by caregivers of patients with epidermolysis bullosa

Question (over the last month)	Answer, n (%)			
	Not at all/not relevant	A little	Quite a lot	Very much
1. Emotional distress (e.g., worry, depression, embarrassment, frustration)	1 (1.3)	4 (5)	18 (22.5)	57 (71.3)
2. Physical well-being (e.g., tiredness, exhaustion, contribution to poor health, sleep/rest disturbance)	1 (1.3)	9 (11.3)	29 (36.3)	41 (51.3)
3. Personal relationships	8 (10)	33 (41.3)	31 (38.8)	8 (10)
4. Problems with other peoples’ reactions due to their relative/partner's skin disease (e.g., bullying, staring, need to explain to others about his/her skin problem)	9 (11.3)	27 (33.8)	35 (43.8)	9 (11.3)
5. Social life (e.g., going out, visiting or inviting people, attending social gatherings)	23 (28.8)	28 (35)	24 (30)	5 (6.3)
6. Recreation/leisure activities (e.g., holidays, personal hobbies, gym, sports, swimming, watching television)	4 (5)	31 (38.8)	32 (40)	13 (16.3)
7. Time spent looking after their relative/partner (e.g., putting on creams, giving medicines, or looking after their skin)?	1 (1.3)	15 (18.8)	26 (32.5)	38 (47.5)
8. Extra housework to do because of their relative/partner's skin disease (e.g., cleaning, vacuuming, washing, cooking)?	0 (0)	6 (7.5)	31 (38.8)	43 (53.8)
9. Affected their job/study (e.g., need to take time off, not able to work, decrease in number of hours worked, problems with people at work)?	7 (8.8)	25 (31.3)	35 (43.8)	13 (16.3)
10. Increased their routine household expenditure (e.g., travel costs, buying special products, creams, cosmetics)?	0 (0)	9 (11.3)	16 (20)	55 (68.8)

The FDLQI was also evaluated based on patient demographic and disease features, as reported in Table 2. The mean number of siblings in each household was 2.5 ± 1.67, and the mean number of patients with EB in each family was 1.16 ± 0.49.

**Table 2:** Quality of life assessment of caregivers of patients with epidermolysis bullosa based on demographic and disease features of the patient

Variable		Frequency, n (%)	FDLQI score mean ± standard deviation	*p*-value*
Sex	Male	47 (58.7)	20.00 ± 5.22	.844
	Female	33 (41.3)	19.79 ± 4.37	
Level of education	None/kindergarten	20 (25)	19.45 ± 5.73	.957
	Primary/secondary School	55 (68.7)	20.14 ± 4.94	
	University	5 (6.3)	20.50 ± 3.21	
Occupation	None	20 (25)	19.45 ± 5.75	.811
	Student	45 (56.3)	20.18 ± 4.53	
	Employed	15 (18.7)	19.53 ± 3.87	
Comorbid conditions	Yes	8 (10)	22.13 ± 4.49	.044
	No	72 (90)	19.63 ± 4.70	
Residence	Urban	34 (42.5)	19.24 ± 5.19	.299
	Rural	46 (57.5)	20.35 ± 4.32	
Subtype of epidermolysis bullosa	Dystrophic	66 (82.5)	20.83 ± 4.08	< .001
	Junctional	2 (2.5)	21.00 ± 0.001	
	Simplex	12 (15)	± 4.78	

FDLQI, Family Dermatology Life Quality Index

⁎ Based on independent sample *t* test or analysis of variance test

Among the factors related to the patient, having a comorbid disease was significantly associated with a higher FDLQI score (*p* = .044). Regarding the subtype of EB in the patients, there was a significant association between the subtype of disease and QoL of the families, with the lowest FDLQI scores (*p* < .001) observed in EB simplex cases. There was also a significant positive association between the number of patients with EB in the family and FDLQI (*r* = 0.185; *p* = .049), indicating that a higher number of patients with EB in a family was associated with higher FDLQI scores. On the other hand, although there was a weak positive correlation between FDLQI score and patient age, this correlation was not statistically significant (*r* = 0.052; *p* = 0.649). There was no significant association between patient age and number of siblings with FDLQI scores (*p* = .325 and .157, respectively).

As demonstrated in Table 3, there was a significant association between a higher degree of education in caregiving mothers and FDLQI scores (*p* < .001). Also, caregiving mothers who were employed had significantly lower FDLQI scores (*p* < .001). With respect to comorbid conditions in caregivers, including diabetes mellitus, hypertension, and chronic heart disease, mothers without comorbid disease had a significantly lower FDLQI score (*p* = .020). Furthermore, the multiple linear regression model, which was conducted with mothers’ education (β = –0.54; *p* ≤ .001), mothers’ occupation (β = –0.04; *p* = .763), and mothers’ comorbidities (β = –0.06; *p* = .575), showed that only mothers’ education could be considered a cofounding factor that affected their FDLQI scores. The results also show that the educational level, occupation, and comorbid diseases of the fathers who participated in this study as caregivers were not significantly associated with their FDLQI score.

**Table 3:** Quality of life assessment of caregivers of epidermolysis bullosa patients based on demographic data and comorbidities of the caregivers

Variable*		Frequency, n (%)	FDLQI score, mean ± standard deviation	*p*-value†
Responder	Father	15 (18.7)	21.87 ± 4.36	.069
	Mother	65 (81.2)	19.42 ± 4.70	
Father`s education	Under high school diploma	3 (20)	21.67± 3.37	.195
	High school diploma and bachelor's degree	7 (46.7)	23.8 ± 5.04	
	Master's degree and above	5 (33.3)	19.20 ± 5.18	
Mother`s education	Under high school diploma	34 (52.3)	21.47 ± 4.13	< .001
	High school diploma and bachelor's degree	21 (32.3)	18.52 ± 3.94	
	Master's degree and above	10 (15.4)	14.30 ± 3.59	
Father`s occupation	Self-employed	6 (40)	21.00 ± 2.75	.825
	Employed	6 (40)	22.00 ± 1.00	
	Retired	3 (20)	22.67 ± 6.59	
Mother`s occupation	Self-employed	4 (6.2)	19.00 ± 5.58	< .001
	Housewife	52 (80)	20.48 ± 3.97	
	Employed	9 (13.8)	13.44 ± 3.90	
Comorbid condition in father	Yes	5 (33.3)	24.20 ± 3.11	.148
	No	10 (66.7)	20.70 ± 4.54	
Comorbid condition in mother	Yes	32 (49.2)	20.78 ± 4.21	.020
	No	33 (50.8)	18.09 ± 4.82	

FDLQI, Family Dermatology Life Quality Index

⁎ The values reported in this table are based on the caregivers who completely filled out the FDLQI questionnaire.

† Based on independent *t* test or analysis of variance test

## Discussion

In this study, we evaluated the FQoL of patients with EB in southern Iran. To the best of our knowledge, this is the first study addressing the FQoL of patients with EB in this region. This study highlights the need to evaluate the QoL indices of this population so that this aspect of the disease can be included in the overall management of this condition. The care of patients with EB is a chronic task; thus, if the FQoL is low, this will certainly affect the constant management and support the patients need. Our survey found that in most families in Iran, mothers stayed home and took care of the child with EB, except those with high-income professions. Moreover, this study revealed that there were remarkable positive associations between better FQoL and a higher level of education in mothers, mothers being employed, patients and mothers having no comorbid disease, the family residing in urban areas, and minor types of EB in the patients.

Another point of interest in the results from this study was that mothers comprised the majority of caregivers (around 80%). Also, approximately half of the mothers had a high school diploma or higher degrees of education, and 80% were housewives. This emphasizes the role of mothers in EB patient care in Iran and the need to initiate and implement specific programs to support them in this endeavor. On the other hand, the low sample size for fathers who participated as caregivers in this study does not accurately depict the parameters involved with fathers as caregivers for patients with EB in Iran.

The FQoL in families with disabled children is often impaired because of several factors, including familial, social, and economic issues (Francisco Mora et al., 2020). Providing care for family members with a chronic disease can be very stressful and time consuming for caregivers and thus impact their quality of life. Furthermore, psychological consequences for both the patient and caregiver may be intensified due to public attitudes toward a noticeable and visible disease (Tabolli et al., 2010). In the case of EB, which is often a serious illness that mainly affects children, caregivers share a heavy burden with the patients (Angelis et al., 2016). The most common issues identified previously by caregivers for patients with EB have been time spent looking after the patient, affected physical well-being, emotional distress, and increased household spending (Sampogna et al., 2013).

Family members play a significant role in providing treatment for the elderly and disabled, as well as providing support (Faison et al., 1999). A review of the literature shows various studies that have evaluated the QoL of caregivers engaged in the care of patients with cancer, schizophrenia, or other diseases (Belasco and Sesso, 2002; Dennis et al., 1998; Magliano et al., 2005); however, not many reports evaluate the family burden of skin diseases (Ferrario et al., 2004). When reviewing the results of issues that exist with respect to the caregivers of patients with EB in our study, the need to offer support interventions to these families is evident and includes relieving the psychological response and stress of relatives in their confrontation with the disease, providing knowledge on the disease course and outcome, training relatives on patient symptom management, and strengthening the social networks of relatives.

Our study showed that caregiving mothers who had a higher education and were employed, as well as those living in urban areas, had a better FQoL score, but only the mother's education was identified as a predicting factor for FDLQI scores. All these factors could be associated with the patient's socioeconomic status and the fact that employed mothers could seek outside help (part-time nurses and cleaning personnel) and pay these costs. Angelis et al. (2016) reported that EB in children affects FQoL through repeated medical visits, hospitalizations, and direct non–health care informal costs (i.e., caregivers' dedication time). Moreover, EB in adults affects FQoL by direct health care costs, especially hospitalizations, repeated medical visits, and health-related materials and equipment, as well as rehabilitation, which represented the vast majority of costs. EB's social/economic burden, shared between the high direct non–health care costs arising from informal care and the loss of labor productivity, reinforces the importance of not limiting the cost analysis to the direct costs of health care (Angelis et al., 2016; Flannery et al., 2020). Future public policy decisions and strategies at a national level for EB and other genetic diseases should seek to consider cost inequalities at the patient level and the impact of health-related QoL. To our knowledge, policies that provide allowances and other benefits to caregivers of patients with EB exist in the United Kingdom, Australia, New Zealand, and possibly other countries (Murrell, 2010).

The Iranian Ministry of Health has also embarked on an initiative to provide necessary dressing materials and various emollient and soothing creams to affected families free of charge. In addition, the EB Home Institute is a registered nongovernmental organization in Iran that helps support patients with EB and their families both socially and financially. Nursing help, the provision of dressings and medications by the government or nongovernmental organizations, and family counseling sessions and psychological support would help boost FQoL.

Based on our results, an important factor associated with the patients' FQoL was the subtype of EB. In our study, EB simplex demonstrated the best FQoL. However, Sampogna et al. (2013) observed that the family burden due to EB was independent of the subtype. A qualitative study on the family burden of EB in children found that the key problems faced by parents were the child being different, the child suffering pain, feelings of uncertainty, difficulties in organization of care, ignorance and lack of skills of care providers, restrictions on employment and leisure time, family problems, never being off-duty, and resistance to difficult care (Dures et al., 2011). According to the results of two recent studies on the various subtypes of EB, the problems were very similar, albeit with different severity and gravity, and they revealed that EB affected several psychological aspects and had mental impacts on patients and their families (Dures et al., 2011; Margari et al., 2010).

There are studies that discuss the association between FQoL and other genodermatoses, as well as autoimmune bullous diseases. The study conducted by Abeni et al. (2021) showed that the symptoms, feelings, and treatment difficulties of Italian patients with autosomal recessive congenital ichthyoses were correlated with the patients’ and their families’ QoL. In addition, the study demonstrated that the psychological and social aspects of the life of patients with autosomal recessive congenital ichthyoses and their families were affected by this disease (Abeni et al., 2021). Moreover, Dufresne et al. (2013) found that therapeutic educational programs in a neutral place (out of routine therapeutic and health care centers) for patients with ichthyoses improved the patients’ and their families’ knowledge and social skills, which could be considered in future studies on Iranian patients with EB. Furthermore, in a study by Sjedianfard et al. (2021) on FQoL in patients with pemphigus vulgaris, FQoL was shown to be poorer in families with male patients, older patients, and shorter duration of disease, as well as in lower-educated caregivers.

There were several limitations in our study. First, this was a single-center study, so we recommend that multicenter studies be designed with larger sample sizes. Second, we did not evaluate patients’ QoL in this study, so we recommend that future studies be conducted to compare patient QoL with their FQoL using the validated QoL EB instrument. Third, our study was conducted with a standard structured questionnaire, but the FQoL of these patients could be evaluated by more items, so we recommend that further qualitative studies be undertaken that could reveal more aspects of FQoL in families of patients with EB. Moreover, the parameters related to the FQoL of fathers as caregivers of patients with EB in this study were not significant. However, as stated previously, this could be due to the low number of fathers who participated in this study. Therefore, we recommend that FQoL be evaluated separately for both the father and mother of each family, irrespective of who is the main caregiver, and compared with each other to obtain better and more comprehensive data.

Comorbidities were checked for each caregiver and the types of comorbidities included were fairly common; hence, excluding caregivers with comorbidities from the study would mean excluding many study participants in this fairly rare disease. Also, we wanted to check FQoL in a real-life setting. In addition, cultural and religious issues and values can also affect QoL and can vary from one place to another; this was not considered in this study. Finally, our study was performed during the COVID-19 pandemic period, and previous studies have shown that COVID-19 has affected the QoL of the Iranian population (Mirzaei et al., 2021; Vahedian-Azimi et al., 2020), so we recommend that this study be repeated after the COVID-19 pandemic to clear any biases. Furthermore, the authors suggest that knowing whether there is any correlation between FDQoL score and EB-specific disease manifestations (e.g., dysphagia due to esophageal strictures or other symptoms) would be of interest in future studies.

## Conclusion

EB is a rare dermatological disease that requires more attention. QoL studies help in understanding the impact of the disease on daily life and provide insights useful for treatment and monitoring. This small-scale study showed that chronic and serious skin diseases, such as EB, could cause a significant burden on the patients' family members. Overall, the care and management of these patients needs adequate psychological support to improve patients’ and their families’ QoL. Families of patients with EB who have a lower socioeconomic status and unemployed caregivers require special attention. Future multicenter studies with a larger sample size are recommended to determine the many parameters these patients and their families face and can help in the design strategies for improvement of their QoL.
